# Robot or human? Manoeuvring switching intention after robot service failure

**DOI:** 10.1371/journal.pone.0333616

**Published:** 2025-11-04

**Authors:** Wing Han Helen Lee, Suk Ha Grace Chan, Binglin Martin Tang, Zhiwen Song

**Affiliations:** 1 Institute of Management and Health, University of Wales Trinity Saint David, Swansea, The United Kingdom; 2 School of Liberal Arts, Macau University of Science and Technology, Macao SAR, China; 3 School of Tourism and History Culture, Chizhou University, Chizhou City, Anhui Province, China; 4 Faculty of International Tourism and Management, City University of Macau, Macao SAR, China; 5 Faculty of Business, City University of Macau, Macao SAR, China; Gannon University, UNITED STATES OF AMERICA

## Abstract

This study attempts to scrutinise tourists’ switching intentions towards human service after a robot service failure, with the zone of tolerance and trust on stance in technology as moderators. The study adopts the unified theory of acceptance and use of technology as a conceptual framework. Quantitative approach through is adopted. Specifically, the retrospective survey is designed to recall robot-service failures,” A total of 330 valid samples were collected and structural equation modelling was employed for data analysis. The findings revealed an insignificant direct effect of tourists’ switching intentions towards human services after a robot service failure. The findings validate the moderating effects of the zone of tolerance and trust stance on technology on tourists’ customer (dis)satisfaction in terms of severity and controllability while denying the reliability aspect. This study fills the literature gap by elucidating paradoxical switching intention and conceptualising work on human–robot behavioural intentions. The study provides theoretical and practical contributions, with limitations and recommendations for future research directions.

## Introduction

Technological innovation has led to the emergence of service robots and has diversified the service horizon across disciplines -. Robot services are technology-driven services for guests—the delivery of services without a human presence. Previous studies primarily emphasised the scope of service robot delivery such as food delivery and check-in/check-out [1]. The appearance and shape of robots, such as human likeliness and customer experience [2–5] played a role in cultivating tourist experiences during service delivery. Previous studies have shed light on tourists’ responses to human and robot services in different scenarios, such as service failure, service recovery, and the pandemic [6]. Specifically, scholars have divergent views on tourist perceptions of robots and human services [7].

Some scholars have noted that perceived value mediates tourists’ switching intention [8]. Others emphasised that cognitive attributes affect tourists’ intentions towards robot services [9]. Others have also stated that anthropomorphic robots facilitate tourists’ intentions to use them [10]. Despite various studies, tourist adoption of robot services has yet to be fully explored [11]. In addition, robot services have shortcomings, and ongoing research has been conducted on human-to-robot switching intentions or adoption [8,12,13]. Studies on tourists’ responses to robot service failures are limited [2]. Specifically, studies on tourists’ switching intentions towards human services after robot service failures are rarely found [7,13,14]. We argue that previous studies have largely focused on positive outcomes instead of placing the lens on service failure and considering the zone of tolerance and trust by users. Our study fills the gaps in switching intentions towards human services and further investigates the moderating effects of the zone of tolerance and trust on the stance in technology under the scenario of robot service failure.

Large studies on customer switching have received considerable attention from academics [15–17]. This study adopts Unified Theory of Acceptance and Use of Technology (UTAUT) as the conceptual framework and employs the Service Robot Integration Willingness Scale as a measurement tool [17] to reflect the complexity of the contemporary dynamics.

The urge to revisit customers’ switching intentions towards human services, the divergence of scholars’ views on robot services and human services, and the need for a contemporary methodology to explain the complexity of tourists’ switching intentions in the tourism and hospitality paradigm [18] are the literature gaps to be bridged. Based on this gap, the present study strives to examine tourists’ switching intentions towards human services in a cognitive scenario of robot service failure. This study adopted a scenario-based approach by proposing a robot service failure scenario and examining the trust stance in technology and the zone of tolerance as moderator. This study provides practical implications for hospitality practitioners to rethink the trust stance of technology and the zone of tolerance from the customer after a service failure. To ensure the performance of a robot service, practitioners must understand the actual performance and consequences of robot service failures. For academic contribution, we combined operations management and service marketing, extended our knowledge, and added value to the literature on service failure by robots. The objectives of this study are as follows:

(i) to understand the relationship between robot service failure and customer satisfaction, which leads to switching intentions towards human service;
(ii) to investigate trust stance in technology as a moderator in the relationship between robot service failure and customer satisfaction; and
(iii) to examine a customer’s zone of tolerance as a moderator in the relationship between customer satisfaction and switching intentions towards human services.

## Literature review

### Paradox of robot and human services

A robot service is a technology-driven service with an incorporated user interface that allows interaction and communication with customers during service delivery A previous study emphasised that technology empowers robot–customer interactions. Over the last decade, academia has had divergent views on robot–human relationships. Some researchers proposed an exclusive relationship, while some mentioned its symbiotic relationship [19,20]. In the realm of symbiosis, robot services take up daily routines and mechanical tasks, while human service is in a place to facilitate customer emotion during the service delivery process in the hospitality context. However, during the COVID-19 period, the customers had a positive attitude towards non-human services. We argue that customers’ switching intention towards human staff service after robot service failure must be scrutinised under the influences of zone of tolerance and trust on stance in technology. Whether customers will attempt to switch to a human service after a robot service mistake exceeds their zone of tolerance remains unclear. This study places the lens on the zone of tolerance and trust on stance in technology as moderator influencing human service intention in the context of robot service failure.

### Robot service failure and switching intention

Service failure is inevitable in the tourism and hospitality industry due to its high service contact in nature and its high level of service heterogeneity [21–24]. Robot service failure refers to the state in which robot service performance falls below customers’ expectation. Robot failures comprise technical and functional failure. These failure(s) would trigger psychological, emotional, and cognitive discomfort such as frustration. [25,26] Additionally, [27] emphasises that robot’s process failure during robot-customer encounters would provoke stronger customers’ emotional discomfort than outcome failures and lead to negative customer dissatisfaction.

Customer satisfaction is strongly related to customers’ switching intention. [26] opined that repeated service failure intensifies customer dissatisfaction and leads to negative behavioural responses, including complaint and switching intention. From an economic point of view, this means that the price paid by customers for a product or service falls short of their expectations. After experiencing a service failure, customers are likely to switch to other products or services to maximise their gain. With this logic of utility maximisation and customers’ rational reasoning, customers are likely to turn to other service providers or suppliers for products or services to maximise their benefits. However, reflecting the complexity of a business paradigm may be too linear. This captures the fact that the price–benefit attribute is not the only factor influencing customers’ switching intentions. Instead, customers’ psychological attributes manipulate their switching intentions. In other words, service failure may not lead to customers’ switching intentions if service recovery optimises or outweighs the negative sentiment incurred by the service failure. The forthcoming would mention “trust in technology” and “zone of tolerance” impact customer switching intention through a theoretical lens.

### Theories

Prospect theory on robot–human interaction and switching intention have been conducted through different lenses, including psychology, economics, and marketing [28,29]. In classical economics, decision-making is based on the assumption of rationality and utility maximisation [30]. Behavioural economics is regarded as a novel methodological approach for elucidating the complexity of customers’ decision-making in reality [31]. [32] suggested heuristic factors influencing customers’ decisions, [33] opined about the role of risk aversion on customers’ consumption.

This study is derived from prospect theory [34]. The prospect theory is a cognitive psychology concept initiated by Kahneman and Tversky in 1979 [35]. The theory features that customers’ behaviour is based upon several principles. First, customers make decisions based on the cost-benefit (value) rule. The theory suggests that customers tend to seek loss aversion rather than value maximisation. Second, customers tend to maintain the status quo because of the framing effect. Switching implies risk and uncertainty, which is cost-taking. With this vein, customers tend to stay with the current state to avoid unforeseen costs induced by switching. Last, customers may be illused when making a decision [36]. Based upon the above principles, the study adopts a retrospective survey to recall customers’ robot failure experience [37,38]. Unified Theory of Acceptance of Use of Technology (UTAUT).

In addition to prospect theory, this study integrates UTAUT to present the relationship between technology and customer acceptance. Perceived ease of use and utility significantly influence users’ willingness to use technology [45, leading to trust-building between technology and humans. The theory construct includes performance expectancy, effort expectancy, social influence, and facilitating conditions, which influence individual behaviour when using technology. A crucial issue lies in the assumption that customers believe that resources and support are available to perform behaviour in technology [39]. We adopted the UTAUT to emphasise the importance of performance expectancy as a predictor of behavioural intention. Customers assess the technology effort associated with the acceptance of the technology. This study extends trust in technology to behavioural economics by recalling the values (benefits) acquired from robot services as a moderator.

### Zone of tolerance (ZOT)

The zone of tolerance is defined as the difference (gap) between the desired level of service level and the minimal acceptable service level [40]. This outlined an acceptable range of outcomes for service delivery [41]. The zone of tolerance is a composite indicator measuring expectation levels, service quality, and customer satisfaction [42]. This difference (gap) reflects the range within which service performance is perceived as acceptable, consistent with the theoretical framework of the ZOT model [43,44].Customer satisfaction is a direct indicator of customers’ switching intention emphasised customers’ positive sentiment usually results in low switching intentions. [45] suggested that tourists’ zone of tolerance is greater during uncertainty to avoid loss. However, studies on the zone of tolerance and switching intention service context have yet to be conducted. This study adopts the zone of tolerance as a moderator that cultivates customers’ thoughts that any service level ranging between acceptable and desired remains positive despite service failure. Nevertheless, any switching intentions can result in negative values.

## Hypothesis development

### Robot service failure affects switching to human staff service

[46] recognised customer switching as a potential behavioural response to robot service failures. Customers encountering robot service failures may proactively opt for human staff service as a resolution. Human staff service is often regarded as the principal alternative when robot service failures occur [47]. Despite the distinct advantages that human employee services offer over their robotic counterparts in various domains [48,49] identified controllability as a pivotal factor that could encourage individuals to forsake robot services in favour of human services. Responsiveness was also a significant factor [50]. Nevertheless, the controllability and responsiveness of robotic services are expected to increase with the evolution of intelligent and human-like robots. Currently, as perceived by customers, these attributes (controllability and responsiveness) lag significantly behind those of human staff services [51].

Therefore, this study proposes the following hypothesis:

H1: Robot service failure positively affects customers’ intention to switch to human staff service.

### Robot service failure affects customer satisfaction

Service failure is defined as a service performance below the expectations of one or more customers [52]. Service failures can lead to customer dissatisfaction [53], negative word-of-mouth propagation [54] and customer switching behaviour [55].

Perceived controllability of service failure refers to the extent to which consumers interpret the causes of failure as controllable or uncontrollable [56]. This involves consumer inferences about whether service providers can prevent or intervene in events that leave them dissatisfied with the service [57]. Humans tend to have a degree of control over their environment to ensure the stability and predictability of their lives [58]. Studies have demonstrated that when consumers have a degree of control over the environment in which they live, their uncertainty and risk perception decrease, and predictability increases [59].

Severity largely depends on the problems caused by failure and consumer perceptions. Failures perceived as serious tend to be negatively associated with satisfaction, trust, and subsequent repurchase behaviour [60–62]. Research has suggested that the higher the severity of a service failure, the greater its impact on customer satisfaction and business outcomes [63]. The more severe the failure, the more difficult it is to recover [64].

A widely used classical definition of reliability is the ability to fulfil promised services accurately and reliably [65]. In previous studies, reliability is usually discussed as one of the dimensions of service quality [66]. Among them, [67] confirmed that all five dimensions of service quality showed a significant correlation with customer satisfaction and customer loyalty, including service reliability. Barua et al. [68] confirmed the effect of customers’ perceived reliability of a self-service technology on their satisfaction. Therefore, this study proposes the following hypotheses:

H2a: The controllability of robot service failure has a significant effect on customer satisfaction.
H2b: The severity of robot service failure has a significant effect on customer satisfaction.
H2c: The reliability of robot service failure has a significant effect on customer satisfaction.

### Customer satisfaction to robotic services negatively affects the intention to switch to human service

Many organisations are currently promoting automated robot services because of the need for big data analytics and the fact that maintaining AI systems is often more cost-effective than using manual equipment [69]. According to the UTAUT, perceived ease of use and utility significantly influence users’ willingness to use technology [70]. Research has shown that when automated services provide quick and relevant feedback, customers tend to favour these automated solutions over interpersonal interactions because of perceived efficiency and convenience [71]. Automated systems provide a consistent quality of service, whereas interpersonal interactions vary according to an individual’s mood, expertise, and other factors. [72] confirmed that the quality of e-services, including AI-powered customer services, has a positive impact on user satisfaction and loyalty, and that high satisfaction with AI customer services is associated with an increased willingness to reuse these services.

However, when ease of use and utility disappear due to service failures caused by robots, customers usually prefer to solve their problems through human interaction. This shift is driven by the need for empathy, problem-solving skills, and personalised services, which automated systems may lack. Therefore, this study proposes the following hypothesis:

H3: Customer satisfaction with robot services negatively affects the intention to switch to human service.

### Customer’s interactive zone of tolerance as a moderator

[73] highlighted three different levels of service quality measures that further quantify the desired service levels, the minimum service level, and customer perception of the actual service. The concept of the zone of tolerance further discusses customers’ level of expectation of service between the desired and adequate services. Many service levels at which customers believe that the firm will actually deliver are referred to as predictable services. However, such customers do not predict the ideal level of expectation but a range of expectations. [74] discussed the range of expectations as the ‘zone of tolerance’, with the desired service at the top and ‘adequate service’ at the bottom of the scale. If the delivered service falls within the zone or is better than the desired level, customers are satisfied. Conversely, if the customer is dissatisfied, their level of expectation is comparable to the tolerable expectations. As mentioned earlier, customers have their level of expectations, ranging from the desired and adequate services. This is referred to as the zone of tolerance and indicates whether a customer can tolerate acceptable satisfaction [75]. Therefore, robot service failure and dissatisfaction may lead to switching behaviour because of the deteriorating service level that falls beyond their zone of tolerance. Thus, we propose the following hypothesis:

H4: A customer’s interactive zone of tolerance serves as a moderator in the relationship between customer satisfaction and intention to switch to human staff service.

### Trust stance in technology as a moderator in the relationship between robot service failure and customer satisfaction

Research has shown that when customers have a high level of trust in a service robot, they are more likely to be satisfied with the overall service [76]. Controllability is a key factor that affects customer attitudes and behaviours when faced with a service failure [77]. When customers perceive a service failure as more controllable, they tend to evaluate service providers negatively. Customers perceive a company as a service provider and are capable of and responsible for preventing or controlling service failures and bringing them back to the expected range. However, if a service provider fails to make it, customers tend to be irritated and blame the service providers in a negative manner. [78] highlighted that service failure severity affects customers’ negative feelings. [78] confirmed the effect of customers’ perceived reliability of self-service in relation to customers’ satisfaction with technology. This suggests that trust in technology can significantly increase customer satisfaction with service robots. By contrast, in service failure scenarios, trust in technology can reduce customers’ negative evaluations of failure and thus maintain high customer satisfaction. Therefore, this study proposes the following hypotheses:

H5a: Trust stance in technology serves as a moderator in the relationship between controllability of robot service failure and customer satisfaction.
H5b: Trust stance in technology serves as a moderator in the relationship between severity of robot service failure and customer satisfaction.
H5c. Trust stance in technology serves as a moderator in the relationship between reliability of robot service failure and customer satisfaction.

**Fig 1:**
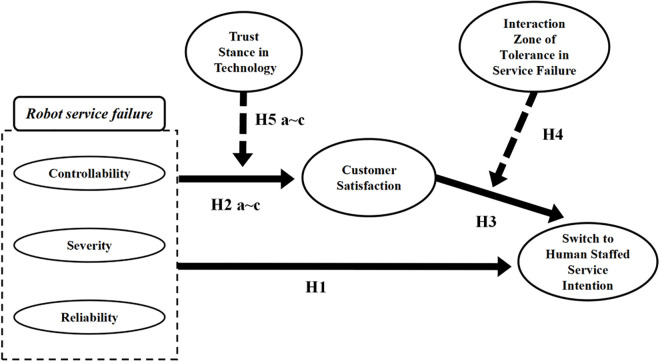
Conceptual model of this research.

Fig 1 illustrates the conceptual model used in this study.

## Methodology

This research adopted a quantitative analysis. The retrospective survey was designed to recall customers’ robot failure experience. A seven-point Likert scale was used for data analysis because of its suitability for construct measurements and in-depth data analysis [79]. In addition, all the scales were adopted from well-established scales. Detailed descriptions of the measurement items and their sources are listed in Table 1. The questionnaire is available in English and Chinese. The authors used the back-translation method to ensure the consistency of both language versions [87].

**Table 1:** Concept and corresponding questionnaire questions.

Construct	Item		*Source*
***Controllability***	CT1	*The robot service failure, they are beyond my sphere of influence.*	*[62,80]*
	CT2	*The robot service* is *virtually not controllable for me.*	
	CT3	*The robot service* is ***easy to control anywhere at any time (reverse).***	
	CT4	*The robot service failure, it can only influences indirectly* ***(reverse)***.	
***Severity***	SE1	*In my opinion, the robot service failure creates big inconvenience.*	*[81]*
	SE2	*In my opinion, the robot service failure creates major aggravation.*	
	SE3	*In my opinion, the robot service failure create minor problem ****(reverse)***.	
***Reliability***	RL1	*When robot service promise to do something by a certain time, they will do so* ***(reverse)***.	*[82,83]*
	RL2	*When customers have a problem, robot service shows a sincere interest in solving it* ***(reverse)***.	
	RL3	*Robot service will perform the service right the first time* ***(reverse)***.	
	RL4	*Robot service will provide their services at the time they promise to do so* ***(reverse)***.	
	RL5	*I think robot service are reliable* ***(reverse)***.	
**Customer satisfaction**	CS1	*I’m satisfied with the quality of the service products available with robot.*	*[84]*
	CS2	*Robot service is a pleasurable experience because it allows me to get a personalized product at my leisure.*	
	CS3	*I tell other folks about purchasing service with robot.*	
**Switch to human-staff service intention**	SW1	*I am rather willingness switch to human-staffed service.*	*[85]*
	SW2	*The likelihood of switch to human-staffed service.*	
	SW3	*The probability that I would consider buying the human -staff service.*	
**Zone of tolerance**	ZOT1	*Robot service have the knowledge to answer customers’ questions.*	*[85]*
	ZOT2	*Robot service instils confidence in customers.*	
	ZOT3	*Customers feel safe in their transactions with the robot service.*	
	ZOT4	*Robot service give prompt service to customers.*	
	ZOT5	*Robot service gives customers individual attention.*	
**Trust stance in Technology**	ST1	*I generally give a technology the benefit of the doubt when I first use it (reverse).*	*[86]*
	ST2	*My typical approach is to trust new technologies until they prove me that I shouldn’t.*	
	ST3	*I usually trust a technology until it gives me a reason not to trust it.*	

*Note: Italic font-dimensions of robot service failure.*

This study was approved by the Human Research Ethics Committee for Non-Clinical Faculties of City University of Macau (reference number: CUM202406003). All procedures involving human participants were conducted in accordance with the ethical standards of the committee and relevant research regulations. Informed written consent was obtained from all participants prior to data collection. At the beginning of the questionnaire, a written Informed Consent form was provided, which detailed the study purpose, questionnaire content, potential risks and benefits, confidentiality guarantees (with emphasis on anonymity), the voluntary nature of participation, and the right to withdraw at any time without penalty. Contact information of the researchers was also included for further inquiries. Participants were required to confirm in the ‘Consent Statement’ that they are of legal age, have read and understood the Informed Consent form, and voluntarily agreed to participate before proceeding to the formal questionnaire sections. All data were collected and analyzed in an anonymous manner to protect participant privacy.

The questionnaire was designed based on a scenario involving robot service failure. The participants were asked to recall their robot service failure scenario and their trust stance in technology and zone of tolerance as a moderator. The questionnaire comprised four sections. It was completed in a self-supervised manner and remained anonymous throughout the entire process. At the beginning of the questionnaire, **Informed Consent** was displayed. Finally, participants must select in the ‘Consent Statement’ that they are adults, confirm their understanding of the Informed Consent, and voluntarily agree to participate before they can proceed to the following section of the questionnaire.

The second section consisted of a definitional statement of robot service failure and a screening question: ‘Have you experienced a robot service failure?’

The third section comprised the construct measurement items. What needs to notice is that:the “zone of tolerance (ZOT)” variable refers to the range of acceptable service performance, bounded by the desired service level (ZOTD) and adequate service level (ZOTA). Adapted from Teas and DeCarlo (2004), we measured ZOTD and ZOTA using 15 items (3 per dimension across reliability, responsiveness, assurance, empathy, and tangibles) on a 9-point scale (1 = very low, 9 = very high). Example items include “The robot service should provide services as promised” (ZOTD) and “Minimum acceptable level for the robot service to provide services as promised” (ZOTA). ZOT was generated by first calculating the average score of ZOTD items (α = 0.80–0.91) and ZOTA items for each respondent, then computing their difference: ZOT = ZOTD (mean of desired items) − ZOTA (mean of adequate items).

Abbreviations are standardized throughout: ZOT (zone of tolerance), ZOTD (desired service level), ZOTA (adequate service level), with non-standard terms (e.g., ZOTE, ZOTL) removed.

While the study adapted measurement items from [85] to fit the robot service context, it extended their approach by explicitly calculating the ZOT gap through the difference between ZOTD and ZOTA. This process ensures that ZOT is not merely a set of items but a quantifiable gap, enhancing the value of the construct as emphasized in the original ZOT model [84].

The fourth part collected demographic information. Part of the reason for including this section is that we intend to use demographic variables as control variables for the final dependent variable, switching intention. This is because factors such as age, the time since the last experience of robot service failure, educational level, and even gender may act as key explanatory variables for switching intention.

Moderating variables including zone of tolerance and trust in stance in technology are deliberately recalled in designated parts with a structured reasoning incorporated. Respondents are reminded to recall their zone of tolerance and their trust on technology when answering the questions.

The sampling was conducted in Macau. Applications of robots in China included but were not limited to telephone customer self-service, hotel check-ins, and financial service recommendations [88–90]. Consumers in China were also highly receptive to and accustomed to the services of non-human employees [91].

Judgmental sampling was adopted in this research. The authors contacted the respondents through social media platforms such as WeChat social media platform. An informed consent form was included at the top of each questionnaire. Before the respondents completed the questionnaire, the authors informed them of the purpose of the research and assured that the data collected would be kept strictly confidential and analysed for academic purposes only. The pilot testing of the questionnaire was conducted from 3–29 January 2025. The number of valid questionnaires collected was 50 for the pilot test and 461 for the formal questionnaire. The official questionnaire collection period was 1–15 March 2025. The total number of questionnaires sent during the formal collection period was 461, but only 330 (71.5%) were valid for data analysis.

Partial least squares structural equation modelling (PLS-SEM) was chosen as the method for analysing data from the formal sample. Compared to CB-SEM, PLS-SEM performs better in small samples with non-normal data. In addition, given the numerous measures in this study, a composite-based model was used in accordance with the recommendations in [92]. Moreover, PLS-SEM was more suitable than CB-SEM for the analysis of the composite-based models [93]. Finally, according to [94], this study used the software platform smartPLS 4.0 for data analysis.

## Data analysis and findings

### Descriptive analysis

Details of the demographic information are presented in Table 2. The descriptive analysis of the data is presented in Table 3. All reverse item values were converted. According to the results in the table, most respondents had experienced a robot service failure within six months (80.6%). The majority of respondents were male (57.6%). The highest percentage of the sample was between the ages of 18 and 30 (74.2%). Nearly half of the participants (44.8%) used robot services between three and five times. Most respondents had obtained a bachelor’s degree or higher (76.7%). Thus, comprehension of the questionnaire should not be a problem for the respondents.

**Table 2:** Data descriptive analysis.

	Mean	Standard deviation	Excess kurtosis	Skewness
**CT1**	3.582	1.131	0.236	0.370
**CT2**	3.736	1.183	−0.349	0.313
**CT3**	3.967	1.179	−0.140	0.009
**SV1**	4.715	1.008	−0.404	−0.315
**SV2**	4.385	0.979	0.068	−0.191
**SV3**	4.718	0.926	−0.324	−0.309
**RL2**	3.615	1.307	−0.076	0.413
**RL3**	3.503	1.079	0.358	0.362
**RL4**	3.076	1.011	0.008	0.290
**RL5**	3.548	1.081	0.539	0.091
**CS1**	4.742	0.807	0.386	0.121
**CS2**	4.721	0.922	−0.444	−0.165
**CS3**	4.721	0.944	0.312	−0.393
**SW1**	2.870	1.049	0.285	0.374
**SW2**	2.867	0.973	−0.320	−0.008
**SW3**	2.664	0.921	−0.731	0.015
**TS1**	4.936	1.098	0.919	−0.785
**TS2**	4.748	1.157	0.174	−0.628
**TS3**	4.652	1.116	0.035	−0.332
**ZOTD1**	5.282	1.417	1.609	−1.374
**ZOTD2**	5.021	1.398	0.590	−0.874
**ZOTD3**	5.073	1.442	−0.050	−0.682
**ZOTD4**	5.606	1.171	2.134	−1.183
**ZOTD5**	4.855	1.554	−0.353	−0.603
**ZOTA1**	4.239	1.563	−0.668	−0.407
**ZOTA2**	4.039	1.389	−0.657	−0.282
**ZOTA3**	4.139	1.586	−0.877	−0.189
**ZOTA4**	4.655	1.496	−0.497	−0.426
**ZOTA5**	3.942	1.657	−0.932	0.024
**ZOT1**	1.100	1.211	1.618	1.227
**ZOT2**	1.003	1.089	2.260	1.283
**ZOT3**	0.985	1.164	1.449	1.212
**ZOT4**	1.012	1.147	1.473	1.175
**ZOT5**	0.979	1.132	1.700	1.213

*Note: CT4 and RL1 are absent because they failed the pilot test; ZOT = ZOTD(Desired service level) – ZOTA(adequate service level)*

**Table 3:** Demographic information.

Demographics	Items	n (%)
**Last time experienced robot failure**	Within one week	56 (17.0%)
	Within one month	111 (33.6%)
	Within half a year	99 (30.0%)
	Within one year	42 (12.7%)
	Over one year	22 (6.7%)
**Gender**	Male	190 (57.6%)
	Female	135 (40.9%)
	Others	5 (1.5%)
**Age Group**	18–30	245 (74.2%)
	31–40	11 (3.3%)
	41–50	24 (7.3%)
	51–60	32 (9.7%)
	over 61	18 (5.5%)
**Frequency of use of robotic services in half a year (times)**	1–2	39 (11.8%)
	3–5	148 (44.8%)
	6–10	76 (23.0%)
	11–15	19 (5.8%)
	Over 15	48 (14.5%)
**Education background**	No formal education	6 (1.8%)
	Junior high school and below	25 (7.6%)
	High School/ Technical secondary school	46 (13.9%)
	Bachelor/ Technical junior college	222 (67.3%)
	Master	23 (7.0%)
	Doctor	8 (2.4%)
**Total**	**/**	**330 (100%)**

### Measurement model

A certainty factor analysis was conducted to explore the reliability and validity of the data. The results of the data reliability tests are presented in Table 4. The factor loadings of all measurement items were above 0.7. This result proves that the measurement items reflected the constructs relatively well. Cronbach’s ɑ and composite reliability exceeded the threshold of 0.7. This result indicates satisfactory internal consistency within the constructs. Average variance extracted (AVE) also exceeded the threshold of 0.5. This result proves the qualified convergent validity of the constructs. Furthermore, results from the PLS-SEM analysis revealed that the outer weights of the three lower-order constructs of robot service failure (RSF) – specifically Controllability, Reliability, and Severity – were 0.480, 0.416, and −0.505, respectively.

**Table 4:** Data reliability and validity.

Construct/Dimension	Item	FL	Cronbach’s ɑ	CR	AVE
***Controllability***	CT1	0.888	0.754	0.759	0.673
	CT2	0.820			
	CT3	0.748			
***Severity***	SV1	0.890	0.910	0.917	0.850
	SV2	0.885			
	SV3	0.987			
***Reliability***	RL2	0.724	0.764	0.781	0.584
	RL3	0.786			
	RL4	0.715			
	RL5	0.827			
**Customer satisfaction**	CS1	0.844	0.785	0.814	0.696
	CS2	0.869			
	CS3	0.788			
**Switch to human-staff service intention**	SW1	0.811	0.798	0.810	0.711
	SW2	0.853			
	SW3	0.864			
**Trust stance in technology**	TS1	0.881	0.843	0.848	0.761
	TS2	0.854			
	TS3	0.882			
**Zone of tolerance**	ZOT1	0.867	0.892	0.922	0.747
	ZOT2	0.891			
	ZOT3	0.881			
	ZOT4	0.816			

*Note: FL- Factor loading; CR- Composite reliability; AVE- Average variance extracted; Italic font- dimensions of robot service failure.*

The results of the discriminant validity test are shown in Table 5. The correlation coefficients of all constructs with other constructs (values outside brackets) are less than the square root of their AVE (bold values). In addition, all HTMT values (values inside brackets) were less than 0.85. This result indicates statistically significant differences between all the constructs.

**Table 5:** Discriminant validity test.

	CS	CT	RL	SV	SW	TS	ZOT
**CS**	**0.834**						
**CT**	−0.328(0.421)	**0.820**					
**RL**	−0.542(0.661)	0.193(0.252)	**0.764**				
**SV**	0.129(0.149)	−0.414(0.501)	−0.153(0.184)	**0.922**			
**SW**	0.254(0.316)	−0.181(0.239)	−0.119(0.173)	0.187(0.214)	**0.843**		
**TS**	−0.344(0.404)	0.227(0.288)	0.226(0.278)	−0.243(0.278)	−0.248(0.304)	**0.872**	
**ZOT**	−0.047(0.099)	0.111(0.160)	0.020(0.089)	−0.042(0.076)	−0.194(0.204)	0.190(0.219)	**0.864**

*Note: Values in brackets are HTMT values; values in bold font and dark coloured areas are square roots of AVE values; the remaining values are correlation coefficients.*

### Structural model

A total of 5,000 bootstrapping iterations were suggested to validate the measurement model [95]. The repeated indicators approach was employed to test the direct relationship between the high-order constructs The results of the tests for H1–H3 are shown in Table 6. Among them, H1 was rejected (β_*RSF → SW*_ = 0.136, p > 0.05). This result suggests that the direct effect of robot service failure on the intention to switch to human service is insignificant. H2a–H2c are supported *(β*_*CT → CS=*_*−0.228, p < 0.001; β*_*SV → CS*_*=−0.125, p < 0.05; β*_*RL → CS*_*=−0.460, p < 0.001)*. This result demonstrates the significant effect of controllability, severity, and reliability on customer satisfaction. H3 is supported *(β_CS → SW_ = 0.212, p < 0.001)*. Therefore, the effect of customer satisfaction on the intention to switch to human staff services was significant. In addition, the influence of control variables on the intention to switch to human service was incorporated into the model. The results indicated that, except for gender, the other three demographic variables (age, education level, and time since the last robot service failure) all exerted a significant impact on the intention to switch to human service.

**Table 6:** Direct effect of PLS-SEM.

	Path	Coefficient	T value	Test result
**H1**	RSF → SW	0.136^ns^	1.959	**Reject**
**H2a**	CT → CS	−0.228^***^	4.440	**Support**
**H2b**	SV → CS	−0.125^*^	2.095	**Support**
**H2c**	RL → CS	−0.460^***^	9.237	**Support**
**H3**	CS → SW	−0.212^***^	3.685	**Support**
	*AGE* - > SW	−0.189^***^	3.723	
	*Gender* - > SW-	0.063^ns^	1.179	
	*Education* - > SW	0.107^*^	2.027	
	*Last time* - > SW	−0.110^*^	2.026	

Note: * p < 0.05; ** p < 0.01; *** p < 0.001, ns – Insignificant effect;the tilted variable is the control variables.

**Fig 2:**
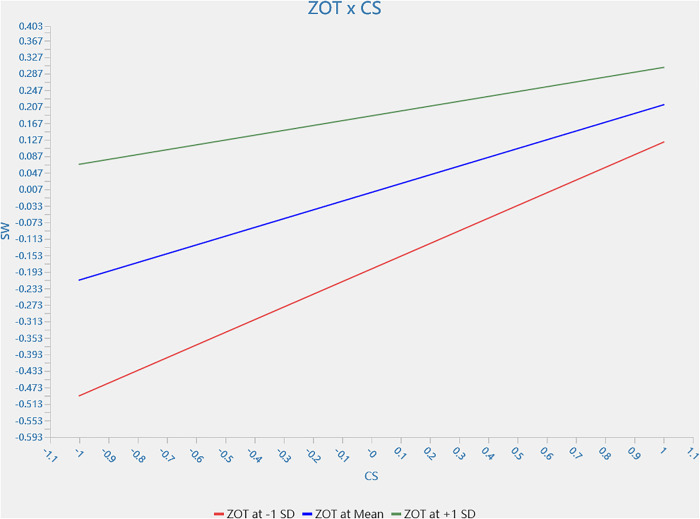
Moderating effect of zone of tolerance on relationship between customer satisfaction and switch to human staff service intention.

According to Table 7, H4 is supported. In other words, customer satisfaction with the intention to switch to human services is moderated by the zone of tolerance. Compared with customers with a high zone of tolerance, customers with a low zone of tolerance are more likely to be motivated to switch to human services because of dissatisfaction (β_ZOT * CS → SW_ = −0.098, 95% CI = [−0.184, − 0.001]). The single slope analysis of H4 is shown in Fig 2,.

**Table 7:** Moderating effect of PLS-SEM.

Hypothesis	Path	Moderating coefficient	Bias	95% CI	Test result
**H4**	ZOT * CS → SW	−0.098	0.004	[-0.184,-0.001]	**Support**
**H5a**	TS * CT → CS	0.121	0.002	[0.020,0.243]	**Support**
**H5b**	TS * SV → CS	0.231	0.000	[0.081,0.397]	**Support**
**H5c**	TS * RL → CS	−0.021	−0.002	[-0.111,0.070]	**Reject**

*Note: CI, confident interval.*

**Fig 3:**
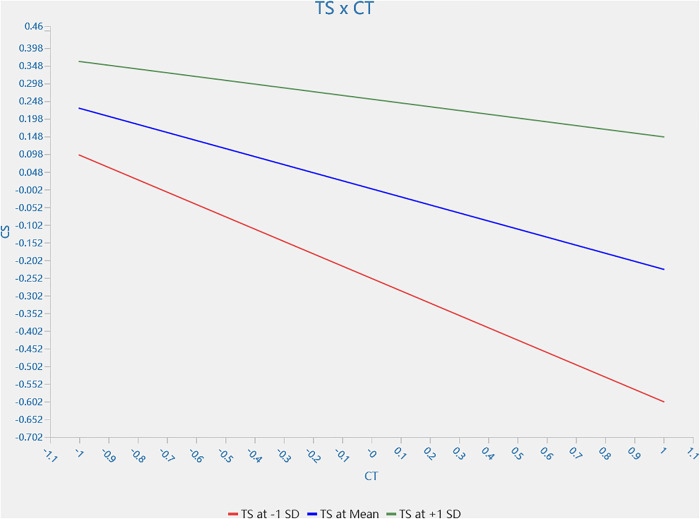
Moderating effect of trust stance in technology on relationship between controllability and customer satisfaction.

**Fig 4:**
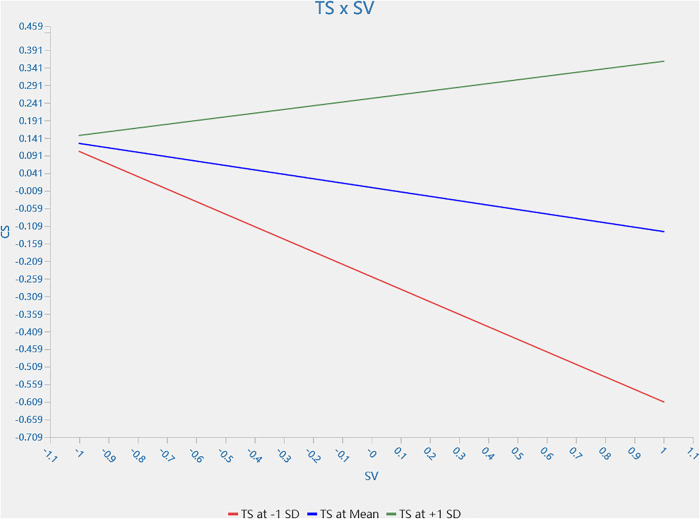
Moderating effect of trust stance in technology on relationship between severity and customer satisfaction.

H5a and H5b were supported, whereas H5c was not. That is, trust stance in technology moderator the effect of controllability (β_TS * CT → CS_ = 0.121, 95% CI = [0.020,0.243]) and severity (β_TS * SV → CS_ = 0.231, 95% CI = [0.081,0.387]) on customer satisfaction. When customer trust in the technology increases, the decreasing effects of controllability and severity on customer satisfaction are mitigated. However, a similar moderating effect of trust stance in technology on reliability is not confirmed (β_TS * RL → CS_= −0.021, 95% CI = [−0.111,0.070]). The single slope analyses of H5a and H5b are shown in Figs 3 and 4, respectively.

The mediating effects of the model are shown in Table 8. Customer satisfaction acts as an intermediate bridge, mediating the significant effects of controllability, severity, and reliability on the intention to switch to human services. In other words, the negative effects of controllability, severity, and reliability on the intention to switch to human services are realised through customer satisfaction.

**Table 8:** Mediating effect of PLS-SEM.

Path	Coefficient	95% CI
CT → CS → SW	−0.048	[-0.089, -0.021]
SV → CS → SW	−0.027	[-0.064, -0.003]
RL → CS → SW	−0.098	[-0.156, -0.046]

According to [96], the threshold for the R^2^ of the endogenous latent variables was 0.19. As illustrated in Table 9, all exogenous variables meet this criterion, as their respective R^2^ values all surpass the specified threshold. Therefore, the variance of the endogenous latent variables in this study’s model can be explained satisfactorily.

**Table 9:** R square of endogenous latent variables.

	CS	SW
**R-square**	0.421	0.196

## Discussion

The current research investigates how robot service failure affects customer satisfaction and contributes to switching intentions towards human service. Findings reveal the insignificant effect of robot service failure on the intention to switch to human service. It is consistent with some literature findings. [25,97,98] while the findings do not support some previous studies [99]. [100] categorise service failure into two dimensions. The first dimension is about basic service failure which is its inability to deliver the basic service and the latter is about the process service failure. For the first dimension, [98] explain that customers are likely to attribute less on robot staff than human staff because customers tend to relate source of failure to the firm instead of the robot. Customer tend to have common understanding that robot are technological hardware and they are prone to errors on some occasions. [101] opinion that service robot failures are regarded as more stable than human failures. For the process service failure, findings (H2a-H2c) further validate that the impact of great controllability, less perceived severity and reliability retains the positive customer satisfaction. The findings support the literature that customers with a greater perceived controllability over the service failure help bring back to the expected range. [102,103]. The findings support the previous study that the positive effect of customers’ perceived reliability of a self-service technology on customer satisfaction [104]

Additionally, the study validates that customers’ positive satisfaction to robot service negatively reduces their switching intention. [105] opinions customers with positive customer satisfaction with robot service is unlikely to switch to human service. The findings imply that the quality of e-services, including AI-powered customer services, has a positive impact on user satisfaction and loyalty, and that high satisfaction with AI customer services is associated with an increased willingness to stay with the robot service despite its failure.

The study further validates the moderator effect of mutual trust with robot staff between robot service failure and customer satisfaction [72]. The findings align with the UTAUT model. The model emphasises that perceived ease of use and utility significantly influence users’ willingness to use technology [106], leading to trust-building between technology and humans. Findings confirm the moderating effect of trust on technology between service failure (controllability and severity) and customer satisfaction. When customer trust in the technology increases, the decreasing effects of controllability and severity on customer satisfaction are mitigated. However, a similar moderating effect of trust stance in technology between reliability and customer satisfaction is not confirmed. The findings are not consistent with the previous study [78, 82,83]. It can be explained that customers do not expect a robot to fix the problem during robot service failure. This implies the value of human staff in the unexpected circumstance emphasising the importance of the robot-staff-human staffed symbiotic relationship during service delivery.

The findings confirm the negative moderating effect of zone of tolerance between customer satisfaction and switch intention to human staff service. The findings are aligned with the literature. [85]. Customers with a low zone of tolerance are more likely to switch to human services because of dissatisfaction. It can be explained by the gain maximization and loss avoidance of prospect theory. Customer satisfaction is decreasing with robot service failure. Despite the moderating effect of the zone of tolerance in which a positive value is still sought, customer satisfaction (value) is decreasing, which may lead to switching intention (loss avoidance).

In summary, the findings address the stated objectives. Specifically, the study provides another perspective on the direct effect of robot failure on switching intention as well as the negative moderating role of trust on the relationship between reliability and customer satisfaction. These findings enrich the academic paradigm and require further exploration.

## Implications

### Theoretical implications

These findings fill the gaps in elucidating customers’ switching intentions by synthesising prospect theory from the contemporary lens of behavioural economics. In addition, this study explains paradoxical behaviour through the moderating effects of the zone of tolerance [40] and the trust stance [70] on customers’ switching intention.

The study has theoretical implications in terms of three aspects. First, the findings provide academia with insights into the relationship between robot service failure and customer intention to switch to human services through changes in customer satisfaction under the influence of the trust stance in technology. This study rejects the direct effect of robot service failure on tourists’ intentions to switch to human services. These findings can be explained by the fact that customers tend to have a positive attitude towards non-human services because of the loss aversion and status quo Conversely, these findings are not consistent with a previous study [99]. Specifically, the findings demonstrate the moderating effect of trust stance in technology (value) and the zone of tolerance on influencing tourists’ intention to switch to human services through the manipulated sequence of questions in the questionnaire, which has not been emphasised by previous studies [107]. Additional research is recommended to further understand the role of moderating effect on tourists’ intention to switch in negative service encounters.

Second, this study exemplifies the moderating effect of the zones of tolerance and trust in technology on tourists’ intentions to switch to human services. It serves as a serious cry out to the academic paradigm on the studies of technology–human relationships or integration. It is our common understanding that a robot-human (staff) is in a symbiotic relationship during the service encounter process. This finding implies that customers who have built mutual trust with robot staff are likely to stay with the robot rather than return to human services despite negative service encounters such as robot service failure. Will robotic services dominate or become part of the norm sooner or later in the fields of tourism and hospitality? As such, ongoing studies on robot–human relationships are required to monitor these changes.

Finally, the findings reveal the moderating effect of the robot trust stance on customer satisfaction in terms of the controllability and severity of robot service failure. However, the findings did not support the moderating effect of the trust stance in technology between reliability and customer satisfaction. That is, trust in robot services in terms of reliability could not moderate customer (dis)satisfaction with robot service failure in the study. This finding may be explained by the fact that reliability is built on the basis of the performance of functional tasks, which are insufficient to influence customer dissatisfaction. Future studies on this topic are recommended.

### Practical implications

The practical implications of this study are two-fold. First, the study indicates that tourists are unlikely to initiate an immediate intention to switch in a robot failure scenario. However, tourist dissatisfaction intensifies with its severity and (un) controllability. As such, a timely revisit of robot–customer service encounters is required to examine customer satisfaction levels. In particular, tremendous effort is required to identify silent customers with some sort of grievance because the accumulated customer dissatisfaction affects the intention to switch in the long run.

Second, the study provides directions for stakeholders and human resource departments in the tourism and hospitality sectors in terms of workforce planning. This study exemplifies the importance of optimising the roles of human and robot services during service delivery. These results support the mitigating effect of controllability and severity on customer satisfaction through an increase in trust in technology. The moderator of trust stance in technology can enhance customer satisfaction through a reminder to customers about the positive values (benefits) that customers can obtain from robot services. This further validates the importance of building trust to retain and increase the number of loyal customers. However, a similar moderating effect of trust stance in technology on reliability was not supported. In other words, an enhanced level of trust in technology cannot safeguard customer satisfaction levels in service failure because the level of reliability is built on the basis of the performance of non-emotional tasks, which is not sufficient to influence customer satisfaction.

Finally, customers with high and low zones of tolerance are more likely to be motivated to switch to human services due to dissatisfaction. Practitioners must pay attention to the proportions of human (human staff) and robot (robot staff) services during service delivery. This study suggests monitoring customer service handled by robot services. It recommends using service staff to facilitate robot service and ensure that they can remedy service deficiencies and follow up after a service failure. These results provide directions for future research on the dimensions of trust cultivated from the functional and emotional aspects.

## Conclusion and limitations

This study has several limitations. First, the study was confined to Macau, which is insufficient for generating findings. Future research can extend this study to different geographical locations to enhance its credibility. Second, this study emphasises customer perspectives, and the results may reflect only one side of the coin. Future research may shed light on employee perspectives to obtain a holistic view. Finally, this study is cross-sectional, and the findings reflect customers’ intentions to switch within a certain period. Similar studies on the intention to switch and human–robot relationships must be conducted in a longitudinal manner. In summary, this study fills a gap in the literature and provides insights into the human–robot relationship in the spectrum of trust stances on technology and customer satisfaction. Moreover, this study enhances the understanding of tourists’ intentions to switch under the scenario of robot service failure, with the moderating effect of the zone of tolerance (loss avoidance) and trust stance in technology in the paradigm of behavioural economics.

## Supporting information

S1 Appendix The original data of this study. (XLSX)
