# Correction: Robot or human? Manoeuvring switching intention after robot service failure

**DOI:** 10.1371/journal.pone.0340212

**Published:** 2026-01-02

**Authors:** Wing Han Helen Lee, Suk Ha Grace Chan, Binglin Martin Tang, Zhiwen Song

The Funding statement for this article is incorrect. The correct Funding statement is as follows: This study received financial support from a university-level grant provided by Macau University of Science and Technology (M.U.S.T.), with the grant number FRG-25-044-SLA.
